# Analysis of Bone Mineral Density/Content of Paratroopers and Hoopsters

**DOI:** 10.1155/2018/6030624

**Published:** 2018-05-20

**Authors:** Yixue Luo, Chenyu Luo, Yuhui Cai, Tianyun Jiang, Tianhong Chen, Wenyue Xiao, Junchao Guo, Yubo Fan

**Affiliations:** ^1^Key Laboratory for Biomechanics and Mechanobiology of Ministry of Education, Beijing Advanced Innovation Centre for Biomedical Engineering, School of Biological Science and Medical Engineering, Beihang University, Beijing 100191, China; ^2^Beijing Advanced Innovation Centre for Biomedical Engineering, Beihang University, Beijing 102402, China; ^3^Beijing Key Laboratory of Rehabilitation Technical Aids for Old-Age Disability, Key Laboratory of Technical Aids Analysis and Identification Key Laboratory of Ministry of Civil Affairs, National Research Centre for Rehabilitation Technical Aids, Beijing 100176, China

## Abstract

The different mechanical stimulus affects the bone mass and bone strength. The aim of this study was to investigate the effect of landing posture of the hoopster and paratrooper on the bone mass. In this study, 39 male participants were recruited including 13 paratroopers, 13 hoopsters, and 13 common students (control groups). Bone area (BA), BMD and BMC of calcaneus, and 1–5th of the metatarsus, hip, and lumbar spine (L_1_–L_4_) were measured by the dual-energy X-ray absorptiometry. Also, the vertical ground reaction forces (GRFs) of hoopsters and paratroopers were measured by the landing of 1.2 m 3D force platform. BA of hoopsters at the calcaneus, lumbar spine, and hip were significantly higher than the control group. The lumbar spine, hip, calcaneus, the 1st and 2nd metatarsals, BMC of paratroopers, and control groups were significantly lower than hoopsters. BMD of the lumbar spine, hip, and right and left femoral necks in hoopsters were significantly higher than the other participants. BMC and BMD of lower limber showed no significant difference between paratroopers and the control group. Besides, peak GRFs of paratroopers (11.06 times of BW) were significantly higher than hoopsters (6.49 times of BW). The higher GRF in the landing train is not always in accordance with higher BMD and BMC. Variable loads in hoopsters can improve bone remodeling and play an important role in bone expansions for trabecular bones. This will be considered by the method of training to prevent bone loss.

## 1. Introduction

Low bone mass, as one of the important factors for osteoporotic fractures, is usually measured with bone mineral content (BMC) and bone mineral density (BMD) [1]. Calcium deficiency, inadequate vitamin D intake, excessive drinking, low reproductive hormone levels, and lack of physical activity are main potential factors of bone loss [2, 3]. It was reported that physical exercise was usually a benefit to increase the bone mass and promote skeletal development [4]. Different loads contribute to bone formation and maintenance of bone metabolism, which also would improve the bone strength or microarchitecture [1, 5, 6]. The mechanical loads of the adult rats showed the difference of intermittent and normal exercises [7].

The cyclic load on bones is generated in different exercises [8]. It was reported that BMD could be increased by duration exercise of more than two hours per week [9]. It was also found that the 2% BMD of the femoral neck was improved by the impact of exercise done for 6 months [10]. Continuous mechanical stimulus helps to maintain bone mass that is important to improve BMD and BMC [11–13]. Osteogenic responses are always produced at the specific loading sites [14, 15]. Basketball, volleyball, and gymnastics from the three times body weight (BW) or greater of reaction force are defined as high-impact exercises [16]. It was beneficial to bone remodeling in high-impact exercises [17]. It was also shown that those high-impact exercises increased BMD and BMC of prepubertal girls [18]. BMD of the total body, lumbar spine (LS), femoral neck (FN), legs, and arms would be increased due to high-impact exercises such as basketball and volleyball exercise [14]. BMC and BMD of the lower limbs were not increased in no-impact or low-impact exercises (cycling and swimming) [19, 20], and there may be not enough stimuli against bones [17].

Basketball exercise included the various postures such as running, starts, stops, and shuffling. The multidirectional loads were produced during exercise. Basketball sport involves mainly jumping and landing [21]. They lean forward with the forefoot landing on the ground first, followed by the whole foot [22]. The twisting movement of the feet was found in balance training in basketball sport [23]. The triceps surae muscle in the musculoskeletal system mainly maintains the stability of ankle joints [24]. The ground reaction forces (GRFs) during the vertical jump-landing were generated in basketball exercise [25]. Meanwhile, parachuting was also a typical high-impact action [26]. Paratroopers perform half-squat parachute landing and keep their feet parallel to ground in the landing process [27]. Dynamic postures of hoopsters and paratroopers were quite different during the landing process. The mechanical loads acting on bones were also different. The effect of different dynamic landing postures on osteogenic responses still needs the quantified research method. In this study, BMD and BMC of hoopsters and paratroopers were investigated by the instruments and experiments, respectively. It would provide suggestion of training methods to prevent bone loss and osteoporotic fracture.

## 2. Methods

Thirty-nine males aged 20–25 years participated in this study (13 paratroopers, 13 hoopsters, and 13 normal men with less involvement in sports as the control group). They were divided into two subgroups: subgroup I with men 20–22 years old including 7 paratroopers, 7 hoopsters, and 7 controls, and the others were subgroup II aged 23–25 years. Volunteers were from the air force base, basketball sports team, and students in university, respectively. Paratroopers and hoopsters participated in training for more than 10 hours weekly compared to less than 1 hour of controls. Height, weight, and body mass index (BMI) of volunteers were shown in Table 1. Each volunteer has no disease of musculoskeletal disorders and bone metabolism.

Bone area (BA, cm^2^), BMD (g/cm^2^), and BMC (g) of the calcaneus, the 1st to the 5th metatarsus, hip (left hip and right hip), and lumbar spine (L_1_–L_4_) were measured by dual-energy X-ray absorptiometry (DXA), respectively. The calcaneus and the metatarsus were placed at 90° inversion and 45° eversion by horizontal scanning of DXA, respectively. The informed consent including the measurement method and the potential risk were signed by volunteers. All measurements were performed in the same condition from March to May, 2016.

The statistical data of three groups (paratroopers, hoopsters, and controls) were compared by the one-way ANOVA test and nonparametric test. The significant differences of BA, BMC, and BMD are shown in Table 1.

**Figure 1:**
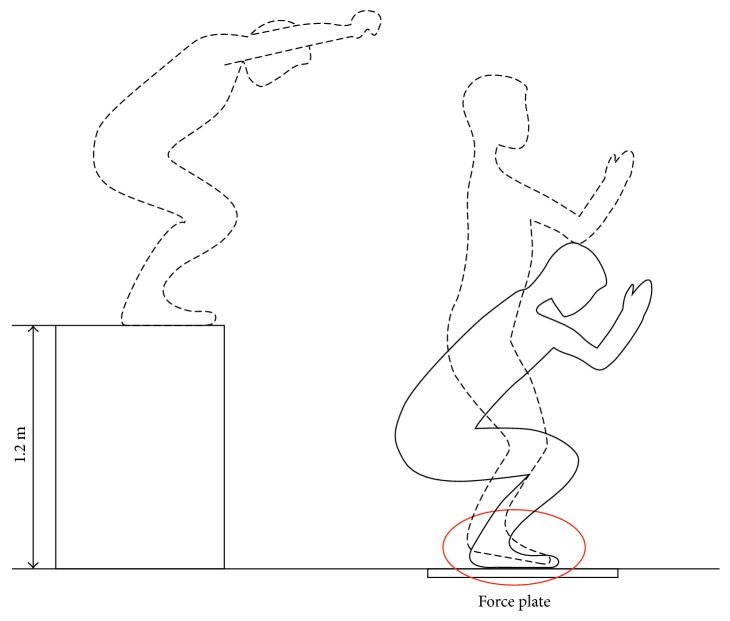
Landing posture of hoopsters.

**Figure 2:**
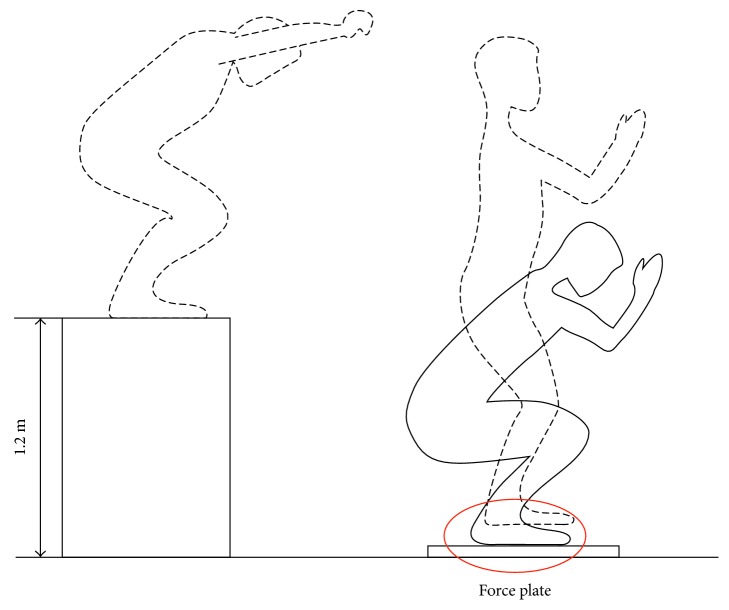
Landing posture of paratroopers.

Besides, hoopsters and paratroopers were required to jump from a 1.2 m platform. The height was is consistent with the velocity of about 6 m/s of paratroopers landing [28]. Landing postures of hoopsters and paratroopers were captured by vidicon (Figures 1 and 2). The GRF was measured by a 3D force platform (1000 Hz, SMA-6, AMTI, USA).

## 3. Results

BA of the calcaneus, metatarsus, hip, femoral neck, and lumbar spine in both groups is shown in Tables 2 and 3. It was found that BA of the calcaneus in hoopsters was significantly larger than controls (*P* < 0.05). The difference of the metatarsal BA among hoopsters, paratroops, and controls was less obvious. In subgroup I, BA of the lumbar spine and hip in hoopsters was significantly greater than controls (*P* < 0.01). Except for the BA of the left and right femoral neck and the fifth metatarsal, hoopsters were significantly greater than paratroopers (*P* < 0.01)

BMC values of the different bones in hoopsters, paratroopers and controls are listed in Tables 4 and 5. BMC of hoopsters' calcaneus and the 1st and 2nd metatarsals was significantly higher than that of paratroopers (*P* < 0.05) and controls (*P* < 0.01). BMC of hoopsters was also significantly higher than controls and paratroopers (*P* < 0.05) at the lumbar spine (L_1_, L_2_, L_3_, L_4_, and total lumbar spine) except for L_3_ in subgroup II. BMC of hoopsters' total hip was the highest compared with controls and paratroopers (*P* < 0.05). However, there was no significant difference among all participants in BMC of the femoral neck.

BMD of the calcaneus in hoopsters was significantly higher than controls (*P* < 0.05) in both groups as shown in Tables 6 and 7. BMD of the first, second, and third metatarsals in hoopsters was significantly greater than controls (*P* < 0.05) in subgroup I. BMD of the third, fourth, and fifth metatarsals in paratroopers was significantly higher than controls (*P* < 0.05) in subgroup II. Higher BMD of the lumbar spine, hip, and femoral neck in hoopsters was obtained statistically compared to other bones (*P* < 0.01). However, paratroopers and controls had no significant difference in BMD at those anatomical locations.

**Figure 3:**
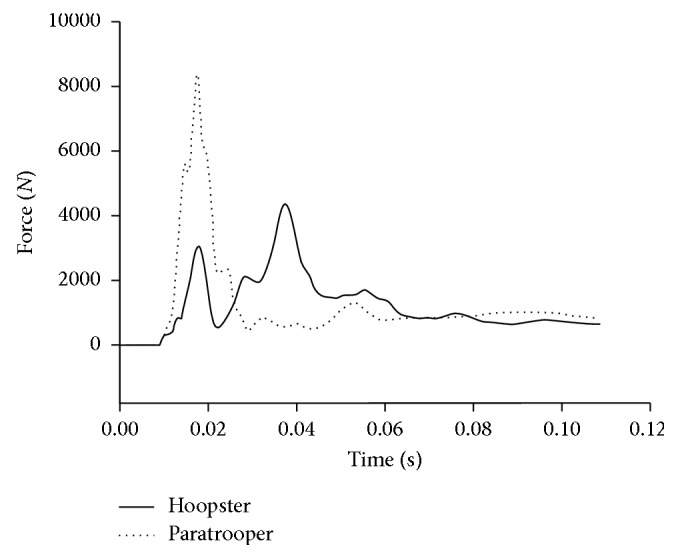
The vertical GRF of hoopsters and paratroopers.

Besides, peak vertical GRFs of paratroopers were 11.06 times of BW (SD ± 0.96) compared to 6.49 times of BW in hoopsters (SD ± 1.19). Compared with the forefoot of hoopsters, which first lands on the ground following the whole foot (Figure 1), the landing posture of paratroopers kept the feet parallel to ground (Figure 2). At the same time, the vertical GRF of both groups are shown in Figure 3. Only one peak value in paratroopers was obtained compared to two peaks of hoopsters.

## 4. Discussion

It was reported that different types of impact exercises including basketball, volleyball, swimming, gymnastics, handball, running, and cycling sports had different effects on BMC and BMD [11, 16, 17]. Basketball sport as a high-impact exercise had positive effect on BMC and BMD [29]. High ground reaction forces were also generated in half-squat parachute landing [26]. Paratroopers kept their feet parallel to ground in the landing process [27]. However, the landing posture of hoopsters was first landing on ground with forefoot, following the whole feet to jump [25]. Dynamic postures of hoopsters and paratroopers were quite different in the landing process. However, the effect of different dynamic landing postures on osteogenic responses still needs the quantified research method. So, the hoopsters and paratroopers as the typical impact subjects were recruited for landing postures to investigate BMD and BMC.

In this study, BMC of the first and second metatarsals in hoopsters was significantly higher than controls. This was consistent with studies that basketball exercising could enhance BMC of the bones [30]. Paratroopers in China perform half-squat parachute landing and keep their feet parallel to ground [27]. Compared with only a peak value of the paratrooper during landing, it was found that the first peak value of GRF was obtained during forefoot of the hoopster first landing on the ground, following the second peak value of the whole feet against ground (Figure 3). It indicated that in daily exercising, jumping of hoopsters with two vertical GRF peaks more effectively generated mechanical loadings at the metatarsals than paratroopers, and this mechanical stimulus would promote local osteogenic responses at loading sites [14, 15]. Thus, land of hoopsters compared to paratroopers will improve BMC of the forefoot after frequent mechanical stimulus. However, it was further proof whether the higher BMC could help paratroopers to reduce injury.

BMC and BMD of the calcaneus and total hip in hoopsters were improved in contrast to controls in our study. It was consistent with previous study that BMC and BMD of the leg, hip, and pelvis were higher than controls [17]. Weight-bearing and high-impact exercises could stimulate bone mineral acquisition in children and adolescents [18, 31]. However, BMC and BMD of the calcaneus and total hip in paratroopers were not sensitive to daily training. It was found that training time of paratroopers in the questionnaire was about 40 to 50 hours weekly, which nearly included 70% of time for landing training. The peak GRF of paratroopers was nearly twice of hoopsters. In our study, it was clear that the high-impact exercising helped with bone formation and enhanced BMD [32]. The effect of the exercise posture on BMD and BMC has the different values for hoopsters' and paratroopers' bones. In Frost's mechanic stability theory, bone mass and bone strengthen were improved with the normal exercise [33]. The peak GRF of paratroopers was about 11 times of BW which would produce excessively large impact force. However, the cyclic loading from basketball exercising may be beneficial to increase BMC and BMD.

Waener et al. [34] found that BMD of all the bones in cyclists, mountain cyclists, was significantly higher. It was shown that the mountain cyclists had varying intensities and frequencies to stimulate osteogenic formation. Similarly, BMD of total lumbar spine and total hip in hoopsters were significantly higher than paratroopers. This was in accordance with the study that the variable velocities in basketball exercising could improve the bone mass and bone strength [22]. Thus, tension, compression, shear, and bending produced at different strain stimulus would act on lumbar spine and hip, which would induce bone formation and enhance BMD at weight-bearing regions [29, 32, 35]. It was also certified that the varying loads could be more benefit to positive osteogenic formation than constant loads [36, 37]. Thus, BMD of the lumbar spine and hip in basketball exercising was higher compared with parachuting. This was in accordance with the study by Platen et al. [38].

BA of the calcaneus, total lumbar spine, and total hip in hoopsters was also significantly higher than controls. This finding was consistent with previous conclusion that the basketball exercising enhanced BA of weight-bearing bones [29]. The BA of the left, right femoral necks and metatarsals in hoopsters was changed mildly compared to controls. It was shown that the mechanical stress of the cortical bone was less sensitive than the trabecular bone [39]. Besides, BA of paratroopers had no promotion compared with controls at measured anatomical locations. Although training of paratroopers was high-impact exercising, it could not generate bone expansions at loaded bones [40]. Different exercise modalities induce variable mechanical stress at stimulated regions [41]. The different BA between paratroopers and hoopsters was caused from the different landing postures.

This study had several limitations. Firstly, the number of paratroopers was limited by air force base. Secondly, there was no dietary information, which may affect bone composition. Thirdly, lean tissue mass and degree of physical fitness were not considered due to the diffcult quantitative methods.

## 5. Conclusions

The high-impact exercises have positive effect on osteogenic formation. BMC and BMD are not in accordance with magnitude of GRF. In this study, basketball exercise from the variable loads may be more effectively increasing BMC and BMD than parachuting with constant loads at loaded sites. Exercising like basketball with high acceleration and multidimensional directions needs further study on its positive effects of bone strength and prevention of osteoporotic fracture caused by bone loss.

## Figures and Tables

**Table 1:** Characteristics of participations.

Variables	Paratroopers^a^	Hoopsters^b^	Controls^c^
*Subgroup I*			
Height (cm)	179.83 ± 4.02	184.29 ± 4.11^a,c^	173.00 ± 6.73
Weight (kg)	70.03 ± 6.32	75.71 ± 8.01	70.66 ± 11.10
BMI (kg·m^−2^)	21.83 ± 1.99	22.29 ± 1.62	23.34 ± 2.68

S*ubgroup II*			
Height (cm)	174.1 ± 3.70	181.17 ± 8.13^a,c^	173.83 ± 2.14
Weight (kg)	68.72 ± 4.80	76.33 ± 8.45	64.33 ± 3.08
BMI (kg·m^−2^)	22.62 ± 2.32	23.18 ± 1.280	21.32 ± 1.23

*Note.* Data are means ± SD; ^a^significantly different with paratroopers, *P* < 0.05; ^b^significantly different with hoopsters, *P* < 0.05; ^c^significantly different with controls, *P* < 0.05.

**Table 2:** BA (cm^2^) of the different anatomical locations in subgroup I.

Variables	Paratroopers^a^ (*n*=7)	Hoopsters^b^ (*n*=7)	Controls^c^ (*n*=7)
Calcaneus	34.49 ± 3.83	36.66 ± 2.61^c^	31.26 ± 3.32
First metatarsal	12.78 ± 0.88	13.54 ± 1.41	11.46 ± 0.74
Second metatarsal	8.37 ± 0.94	9.04 ± 1.28^c^	7.76 ± 0.85
Third metatarsal	7.79 ± 1.13	8.27 ± 0.98	7.30 ± 0.70
Fourth metatarsal	7.95 ± 0.83	8.41 ± 1.15	7.42 ± 0.87
Fifth metatarsal	10.85 ± 1.14	10.24 ± 1.35	9.73 ± 1.28
Lumbar spine (L_1_–L_4_) L_1_	13.97 ± 1.70	16.43 ± 1.05^a,c^	13.96 ± 0.83
L_2_	15.39 ± 0.10	17.50 ± 2.06^a,c^	14.84 ± 1.17
L_3_	17.16 ± 1.31	19.09 ± 2.74^c^	16.54 ± 1.31
L_4_	18.67 ± 1.15	22.54 ± 2.39^a,c^	18.17 ± 2.15
L_total_	65.19 ± 4.40	75.56 ± 7.56^a,c^	63.51 ± 4.52
Left femoral neck	6.10 ± 1.50	5.64 ± 0.38	5.64 ± 0.37
Left hip	40.78 ± 3.35	47.11 ± 4.14^a,c^	39.64 ± 3.77
Right femoral neck	6.77 ± 1.30^b,c^	5.46 ± 0.42	5.45 ± 0.43
Right hip	42.79 ± 3.0	46.53 ± 3.95^c^	38.46 ± 4.89

*Note*. Data are means ± SD; ^a^significantly different with paratroopers, *P* < 0.05; ^b^significantly different with hoopsters, *P* < 0.05; ^c^significantly different with controls, *P* < 0.05.

**Table 3:** BA (cm^2^) of the different anatomical locations in subgroup II.

Variables	Paratroopers^a^ (*n*=6)	Hoopsters^b^ (*n*=6)	Controls^c^ (*n*=6)
Calcaneus	32.00 ± 2.79	37.50 ± 4.71^a,c^	31.95 ± 2.85
First metatarsal	12.59 ± 1.58	13.40 ± 2.88	11.55 ± 1.46
Second metatarsal	8.30 ± 0.81	8.86 ± 1.06	8.11 ± 0.51
Third metatarsal	6.91 ± 0.69	7.93 ± 1.10	7.22 ± 0.44
Fourth metatarsal	7.08 ± 0.49	7.94 ± 0.48	7.42 ± 0.77
Fifth metatarsal	9.54 ± 0.48	10.57 ± 1.01	9.17 ± 1.08
Lumbar spine (L_1_–L_4_) L_1_	14.19 ± 0.95	15.38 ± 1.43	14.26 ± 1.19
L_2_	15.16 ± 1.18	16.36 ± 1.48	15.32 ± 0.49
L_3_	17.17 ± 2.12	18.12 ± 1.52	16.27 ± 1.63
L_4_	18.10 ± 0.98	19.78 ± 2.00^a^	18.30 ± 0.63
L_total_	64.61 ± 4.83	69.64 ± 6.27	64.15 ± 3.18
Left femoral neck	5.15 ± 1.32	5.27 ± 1.17	5.35 ± 0.26
Left hip	41.21 ± 4.19	44.07 ± 4.28	40.72 ± 2.17
Right femoral neck	5.21 ± 1.14	5.78 ± 0.76	4.99 ± 0.46
Right hip	41.57 ± 3.03	45.03 ± 4.12	40.91 ± 3.26

*Note*. Data are means ± SD; ^a^significantly different with paratroopers, *P* < 0.05; ^b^significantly different with hoopsters, *P* < 0.05; ^c^significantly different with controls, *P* < 0.05.

**Table 4:** BMC (g) of the different anatomical locations in subgroup I.

	Paratroopers^a^ (*n*=7)	Hoopsters^b^ (*n*=7)	Controls^c^ (*n*=7)
Calcaneus	27.84 ± 3.89	32.52 ± 5.12^a,c^	24.58 ± 2.64
First metatarsal	6.19 ± 0.85	8.01 ± 1.84^a,c^	5.68 ± 0.78
Second metatarsal	3.54 ± 0.36	4.60 ± 0.84^a,c^	3.15 ± 0.58
Third metatarsal	3.09 ± 0.38	3.61 ± 1.15^c^	2.41 ± 0.38
Fourth metatarsal	2.90 ± 0.60	2.96 ± 0.79	2.26 ± 0.48
Fifth metatarsal	3.82 ± 0.86	3.59 ± 0.81	3.06 ± 0.73
Lumbar spine (L_1_–L_4_) L_1_	13.34 ± 1.68	17.29 ± 1.31^a,c^	13.13 ± 1.87
L_2_	15.43 ± 1.43	19.70 ± 2.06^a,c^	15.08 ± 2.52
L_3_	17.79 ± 1.48	23.03 ± 3.34^a,c^	17.56 ± 2.26
L_4_	19.19 ± 0.93	25.31 ± 2.31^a,c^	19.42 ± 2.49
L_total_	65.75 ± 5.16	85.33 ± 8.38^a,c^	65.19 ± 8.55
Left femoral neck	5.84 ± 1.33	6.86 ± 0.92	5.41 ± 0.78
Left hip	40.51 ± 5.09	58.94 ± 5.05^a,c^	41.62 ± 3.00
Right femoral neck	6.46 ± 1.34	6.72 ± 0.72	5.18 ± 0.67^b^
Right hip	41.96 ± 4.35	57.45 ± 4.61^a,c^	40.36 ± 5.76

*Note*. Data are means ± SD; ^a^significantly different with paratroopers, *P* < 0.05; ^b^significantly different with hoopsters, *P* < 0.05; ^c^significantly different with controls, *P* < 0.05.

**Table 5:** BMC (g) of the different anatomical locations in subgroup II.

Variables	Paratroopers^a^ (*n*=6)	Hoopsters^b^ (*n*=6)	Controls^c^ (*n*=6)
Calcaneus	23.85 ± 2.98	32.95 ± 9.03^a,c^	22.82 ± 3.15
First metatarsal	6.46 ± 0.81	7.59 ± 1.88^a,c^	5.64 ± 0.93
Second metatarsal	3.55 ± 0.43	4.22 ± 0.73^a,c^	3.34 ± 0.36
Third metatarsal	2.67 ± 0.50	2.61 ± 0.88	2.17 ± 0.96
Fourth metatarsal	2.65 ± 0.39^c^	2.50 ± 0.64	2.01 ± 0.29
Fifth metatarsal	3.43 ± 0.55	3.51 ± 0.89	2.57 ± 0.47
Lumbar spine (L_1_–L_4_) L_1_	14.19 ± 1.06	17.20 ± 2.97^a,c^	12.49 ± 1.47
L_2_	15.74 ± 1.02	19.84 ± 3.83^a,c^	14.02 ± 1.01
L_3_	18.93 ± 2.27	22.50 ± 4.24^c^	14.88 ± 1.95^a,b^
L_4_	19.16 ± 2.57	23.70 ± 4.22^a,c^	16.09 ± 1.45
L_total_	68.02 ± 6.46	83.24 ± 14.95^a,c^	57.47 ± 4.81
Left femoral neck	4.89 ± 1.73	6.29 ± 1.94	4.51 ± 0.67
Left hip	40.86 ± 7.54	53.28 ± 7.56^a,c^	38.67 ± 5.12
Right femoral neck	5.36 ± 1.63	6.73 ± 1.51^c^	4.14 ± 0.39
Right hip	42.96 ± 7.42	53.68 ± 7.50^a,c^	38.44 ± 4.88

*Note*. Data are means ± SD; ^a^significantly different with paratroopers, *P* < 0.05; ^b^significantly different with hoopsters, *P* < 0.05; ^c^significantly different with controls, *P* < 0.05.

**Table 6:** BMD (g/cm^2^) of the different anatomical locations in subgroup I.

Variables	Paratroopers^a^ (*n*=7)	Hoopsters^b^ (*n*=7)	Controls^c^ (*n*=7)
Calcaneus	0.81 ± 0.94	0.88 ± 0.92^c^	0.79 ± 0.48
First metatarsal	0.49 ± 0.66	0.59 ± 0.85^a,c^	0.50 ± 0.57
Second metatarsal	0.43 ± 0.44	0.51 ± 0.64^c^	0.40 ± 0.47
Third metatarsal	0.40 ± 0.75	0.43 ± 0.95^c^	0.33 ± 0.33
Fourth metatarsal	0.37 ± 0.08	0.35 ± 0.05	0.30 ± 0.05
Fifth metatarsal	0.36 ± 0.09	0.35 ± 0.04	0.31 ± 0.05
Lumbar spine (L_1_–L_4_) L_1_	0.96 ± 0.04	1.05 ± 0.05	0.94 ± 0.10
L_2_	1.00 ± 0.05	1.13 ± 0.03	1.01 ± 0.10
L_3_	1.04 ± 0.06	1.21 ± 0.04^a,c^	1.06 ± 0.10
L_4_	1.03 ± 0.04	1.13 ± 0.09	1.07 ± 0.06
L_total_	1.01 ± 0.04	1.13 ± 0.04^a,c^	1.02 ± 0.08
Left femoral neck	0.96 ± 0.08	1.21 ± 0.13^a,c^	0.96 ± 0.10
Left hip	0.99 ± 0.08	1.25 ± 0.09^a,c^	1.05 ± 0.09
Right femoral neck	0.95 ± 0.11	1.23 ± 0.08^a,c^	0.95 ± 0.09
Right hip	0.98 ± 0.08	1.24 ± 0.07^a,c^	1.05 ± 0.07

*Note*. Data are means ± SD; ^a^significantly different with paratroopers, *P* < 0.05; ^b^significantly different with hoopsters, *P* < 0.05; ^c^significantly different with controls, *P* < 0.05.

**Table 7:** BMD (g/cm^2^) of the different anatomical locations in subgroup II.

Variables	Paratroopers^a^ (*n*=6)	Hoopsters^b^ (*n*=6)	Controls^c^ (*n*=6)
Calcaneus	0.75 ± 0.13	0.87 ± 0.14^c^	0.71 ± 0.07
First metatarsal	0.52 ± 0.09	0.56 ± 0.03^c^	0.49 ± 0.05
Second metatarsal	0.43 ± 0.08	0.47 ± 0.04	0.41 ± 0.02
Third metatarsal	0.39 ± 0.07	0.33 ± 0.08	0.30 ± 0.01^a^
Fourth metatarsal	0.38 ± 0.06	0.31 ± 0.07	0.27 ± 0.03^a^
Fifth metatarsal	0.36 ± 0.07	0.33 ± 0.07	0.28 ± 0.03^a^
Lumbar spine (L_1_–L_4_) L_1_	1.00 ± 0.08	1.11 ± 0.12	0.88 ± 0.07
L_2_	1.04 ± 0.11	1.20 ± 0.15^a,c^	0.91 ± 0.04
L_3_	1.11 ± 0.13	1.23 ± 0.15	0.91 ± 0.06
L_4_	1.06 ± 0.15	1.19 ± 0.11	0.88 ± 0.08
L_total_	1.06 ± 0.12	1.19 ± 0.13^a,c^	0.90 ± 0.05
Left femoral neck	0.94 ± 0.15	1.18 ± 0.15^a,c^	0.85 ± 0.14
Left hip	0.99 ± 0.14	1.21 ± 0.10^a,c^	0.95 ± 0.10
Right femoral neck	1.02 ± 0.18	1.16 ± 0.15^a,c^	0.84 ± 0.10
Right hip	1.03 ± 0.17	1.19 ± 0.09^a,c^	0.94 ± 0.08

*Note*. Data are means ± SD; ^a^significantly different with paratroopers, *P* < 0.05; ^b^significantly different with hoopsters, *P* < 0.05; ^c^significantly different with controls, *P* < 0.05.

## References

[1] Wolff I., Van Croonenborg J. J., Kemper H. C. G., Kostense P. J., Twisk J. W. R. (1999). The effect of exercise training programs on bone mass: a meta-analysis of published controlled trials in pre-and postmenopausal women. *Osteoporosis International*.

[2] Bakhireva L. N., Barrett-Connor E., Kritz-Silverstein D., Morton D. J. (2004). Modifiable predictors of bone loss in older men: a prospective study. *American Journal of Preventive Medicine*.

[3] Olszynski W. P., Davison K. S., Adachi J. D. (2004). Osteoporosis in men: epidemiology, diagnosis, prevention, and treatment. *Clinical Therapeutics*.

[4] Nikander R., Sievänen H., Heinonen A. (2010). Targeted exercise against osteoporosis: a systematic review and meta-analysis for optimising bone strength throughout life. *BMC Medicine*.

[5] Francis S. L., Letuchy E. M., Levy S. M., Janz K. F. (2014). Sustained effects of physical activity on bone health: Iowa Bone Development Study. *Bone*.

[6] Borer K. T. (2005). Physical activity in the prevention and amelioration of osteoporosis in women. *Sports Medicine*.

[7] Shirazi-Fard Y., Metzger C. E., Kwaczala A. T., Judex S., Bloomfield S. A., Hogan H. A. (2014). Moderate intensity resistive exercise improves metaphyseal cancellous bone recovery following an initial disuse period, but does not mitigate decrements during a subsequent disuse period in adult rats. *Bone*.

[8] Marques E. A., Wanderley F., Machado L. (2011). Effects of resistance and aerobic exercise on physical function, bone mineral density, OPG and RANKL in older women. *Experimental Gerontology*.

[9] McKay H. A., Petit M. A., Schutz R. W., Prior J. C., Barr S. I., Khan K. M. (2000). Augmented trochanteric bone mineral density after modified physical education classes: a randomized school-based exercise intervention study in prepubescent and early pubescent children. *Journal of Pediatrics*.

[10] Bailey C. A., Brooke-Wavell K. (2010). Optimum frequency of exercise for bone health: randomised controlled trial of a high-impact unilateral intervention. *Bone*.

[11] Rector R. S., Rogers R., Ruebel M., Hinton P. S. (2008). Participation in road cycling vs running is associated with lower bone mineral density in men. *Metabolism*.

[12] Allison S. J., Folland J. P., Rennie W. J., Summers G. D., Brooke-Wavell K. (2013). High impact exercise increased femoral neck bone mineral density in older men: a randomised unilateral intervention. *Bone*.

[13] Kelley G. A., Kelley K. S., Kohrt W. M. (2013). Exercise and bone mineral density in men: a meta-analysis of randomized controlled trials. *Bone*.

[14] Duncan C. S., Blimkie C. J., Cowel C. T., Burke S. T., Briody J. N., Howman-Giles R. (2002). Bone mineral density in adolescent female athletes: relationship to exercise type and muscle strength. *Medicine and Science in Sports and Exercise*.

[15] Nurmi-Lawton J. A., Baxter-Jones A. D., Mirwald R. L. (2004). Evidence of sustained skeletal benefits from impact-loading exercise in young females: a 3-year longitudinal study. *Journal of Bone and Mineral Research*.

[16] Milanese C., Piscitelli F., Cavedon V., Zancanaro C. (2014). Effect of distinct impact loading sports on body composition in pre-menarcheal girls. *Science & Sports*.

[17] Ubago-Guisado E., Gómez-Cabello A., Sánchez-Sánchez J., García-Unanue J., Gallardo L. (2015). Influence of different sports on bone mass in growing girls. *Journal of Sports Sciences*.

[18] Courteix D., Lespessailles E., Peres S. L., Obert P., Germain P., Benhamou C. L. (1998). Effect of physical training on bone mineral density in prepubertal girls: a comparative study between impact-loading and non-impact-loading sports. *Osteoporosis International*.

[19] Rizzoli R., Bianchi M. L., Garabedian M., McKay H. A., Moreno L. A. (2010). Maximizing bone mineral mass gain during growth for the prevention of fractures in the adolescents and the elderly. *Bone*.

[20] Maïmoun L., Coste O., Philibert P. (2013). Peripubertal female athletes in high-impact sports show improved bone mass acquisition and bone geometry. *Metabolism*.

[21] Ziv G., Lidor R. (2010). Vertical jump in female and male volleyball players: a review of observational and experimental studies. *Scandinavian Journal of Medicine & Science in Sports*.

[22] Crisafulli A., Melis F., Tocco F., Laconi P., Lai C., Concu A. (2002). External mechanical work versus oxidative energy consumption ratio during a basketball field test. *Journal of Sports Medicine and Physical Fitness*.

[23] McGuine T. A., Keene J. S. (2006). The effect of a balance training program on the risk of ankle sprains in high school athletes. *American Journal of Sports Medicine*.

[24] Zech A., Hubscher M., Vogt L., Banzer W., Hänsel F., Pfeifer K. (2010). Balance training for neuromuscular control and performance enhancement: a systematic review. *Journal of Athletic Training*.

[25] Dook J. E., James C., Henderson N. K., Price R. I. (1997). Exercise and bone mineral density in mature female athletes. *Medicine and Science in Sports and Exercise*.

[26] Whitting J. W., Steele J. R., Jaffrey M. A., Munro B. J. (2007). Parachute landing fall characteristics at three realistic vertical descent velocities. *Aviation, Space, and Environmental Medicine*.

[27] Niu W., Wang Y., He Y., Fan Y., Zhao Q. (2010). Biomechanical gender differences of the ankle joint during simulated half-squat parachute landing. *Aviation, Space, and Environmental Medicine*.

[28] Cilli F., Mahirogullar M., Inan M. (2006). Parachuting injuries: a retrospective study of 43,690 military descents. *Balkan Military Medical Review*.

[29] Falcai M. J., Zamarioli A., Okubo R., de Paula F. J. A., Volpon J. B. (2015). The osteogenic effects of swimming, jumping, and vibration on the protection of bone quality from disuse bone loss. *Scandinavian Journal of Medicine & Science in Sports*.

[30] Zribi A., Zouch M., Chaari H. (2014). Enhanced bone mass and physical fitness in prepubescent basketball players. *Journal of Clinical Densitometry*.

[31] Hind K., Burrows M. (2007). Weight-bearing exercise and bone mineral accrual in children and adolescents: a review of controlled trials. *Bone*.

[32] Creighton D. L., Morgan A. L., Boardley D., Gunnar Brolinson P. (2001). Weight-bearing exercise and markers of bone turnover in female athletes. *Journal of Applied physiology*.

[33] Frost H. M. (1990). Skeletal structural adaptations to mechanical usage (SATMU): 1. Redefining Wolff’s law: the bone modeling problem. *The Anatomical Record*.

[34] Warner S. E., Shaw J. M., Dalsky G. P. (2002). Bone mineral density of competitive male mountain and road cyclists. *Bone*.

[35] Yang P., Brüggemann G. P., Rittweger J. (2011). What do we currently know from in vivo bone strain measurements in humans?. *Journal of Musculoskeletal and Neuronal Interactions*.

[36] Hull M. L. (1983). Analysis of road induced loads in bicycle frames. *Journal of Mechanisms, Transmissions, and Automation in Design*.

[37] Wang E. L., Hull M. L. (1997). A dynamic system model of an off-road cyclist. *Journal of Biomechanical Engineering*.

[38] Platen P., Chae E., Antz R., Lehmann R., Kühlmorgen J., Allolio B. (2001). Bone mineral density in top level male athletes of different sports. *European Journal of Sport Science*.

[39] Chaari H., Zouch M., Zribi A., Bouajina E., Zaouali M., Tabka Z. (2013). Specific sites of bone expansion depend on the level of volleyball practice in prepubescent boys. *Biology of Sport*.

[40] Zouch M., Jaffré C., Thomas T. (2008). Long-term soccer practice increases bone mineral content gain in prepubescent boys. *Joint Bone Spine*.

[41] Zouch M., Chaari H., Zribi A. (2016). Volleyball and basketball enhanced bone mass in prepubescent boys. *Journal of Clinical Densitometry*.

