# Erratum to “Analysis of Bone Mineral Density/Content of Paratroopers and Hoopsters”

**DOI:** 10.1155/2021/6787164

**Published:** 2021-02-12

**Authors:** Luo Yixue, Chenyu Luo, Yuhui Cai, Tianyun Jiang, Chen Tianhong, Xiao Wenyue, Junchao Guo, Yubo Fan

**Affiliations:** ^1^Key Laboratory for Biomechanics and Mechanobiology of Ministry of Education, Beijing Advanced Innovation Centre for Biomedical Engineering, School of Biological Science and Medical Engineering, Beihang University, Beijing 100191, China; ^2^Beijing Advanced Innovation Centre for Biomedical Engineering, Beihang University, Beijing 102402, China; ^3^Beijing Key Laboratory of Rehabilitation Technical Aids for Old-Age Disability, Key Laboratory of Technical Aids Analysis and Identification Key Laboratory of Ministry of Civil Affairs, National Research Centre for Rehabilitation Technical Aids, Beijing 100176, China

In the article titled “Analysis of Bone Mineral Density/Content of Paratroopers and Hoopsters” [1], there was an error in corresponding author, where “Yubo Fan” should be corrected to “Yubo Fan and Junchao Guo.”

The corrected corresponding authors are shown in the corresponding author line above.
